# Dietary Pattern, Nutrition-Related Knowledge and Attitudes of Working Women in Western Province, Sri Lanka

**DOI:** 10.3390/nu15133007

**Published:** 2023-06-30

**Authors:** Ayesha Salwathura, Faruk Ahmed

**Affiliations:** 1 Sri Lanka Standards Institution, 17, Victoria Place, Elvitigala Mawatha, Colombo 00800, Sri Lanka; arsalwathura@gmail.com; 2 Public Health, School of Medicine and Dentistry, Gold Coast Campus, Griffith University, Gold Coast, QLD 4220, Australia

**Keywords:** dietary pattern, dietary diversity score, nutrition knowledge, nutrition attitudes

## Abstract

Healthy eating behaviour of women is critical not only for their health but also for their children’s health and well-being. The present study examined the dietary pattern, nutrition-related knowledge, and attitudes of working women in Western Province, Sri Lanka. In addition, this study identified the factors associated with dietary diversity score (DDS). A cross-sectional study was conducted among 300 working women, aged 20–60 years, in Western Province, Sri Lanka. The data on socio-demography, dietary patterns, and nutrition-related knowledge and attitudes were collected. Overall, 38% of the women were overweight and 13% were obese. The median frequency of intake of chicken, fish, eggs, milk and milk products, green leafy vegetables, and fruits were 2, 5, 2, 9, 5, and 10 respectively, per week. A large majority of the women (70%) had tea/coffee with sugar and snacks (60%) at least four times a week. Only a third of the women met the minimum DDS, while more than half of the women had good nutrition-related knowledge and attitudes. Women with good nutrition-related knowledge were more frequent consumers of roots/tubers, shellfish, vegetables, fruit, fruit juice, nuts and oils, and fast food. Women with good nutrition-related attitudes had a significantly lower frequency of consumption of soya meat, while having a higher frequency of consumption of fast food. Multiple regression analysis showed that age and household income were significantly independently related to DDS, while attitudes were negatively associated. While there was a trend, the association of nutrition-related knowledge with DDS was not statistically significant (*p* = 0.057). The overall F ratio (8.46) was highly significant (*p* = 0.001) and the adjusted R^2^ was 0.093. The results demonstrate that a significant proportion of working women have good basic nutrition-related knowledge and attitudes, while two-thirds of them do not meet the minimum DDS. Furthermore, age, family income, and knowledge were positively associated with DDS, while attitudes were negatively associated. Before designing any intervention, further research is needed using a qualitative approach to understand how nutrition knowledge and eating behaviour are related in this population group.

## 1. Introduction

In recent years, women are increasingly taking advantage of opportunities in the job market because of increased levels of education and a greater necessity to supplement household income. In Sri Lanka, the employment rate for women is 92.1%, with nearly half of them (46.7%) engaged in the service sector and the rest working in industry and agriculture [1]. However, the changed social status of women has resulted in additional workload and stress which may lead to adverse effects on the health and nutritional status of working women. There is increasing concern that working women may contribute to the growing burden of non-communicable diseases such as high blood pressure, diabetes, cardiovascular diseases, cancer, etc. [2,3]. In Sri Lanka, about 24% of working-age women have been suffering from various chronic diseases [2]. In a recent review, Idris et al. [3] reported that low physical activity, a sedentary lifestyle, and poor dietary habits such as skipping breakfast, frequent snacking, junk-food consumption, and low intake of vegetables and fruits are the most common risk factors of developing non-communicable diseases among working women. Malnutrition in women can significantly affect their participation in the job market and their productivity, which in turn leads to economic losses for families and societies [4,5]. In addition, adequate nutritional status of women is critical not only for their health but also for their children’s health and well-being [6,7,8].

While there is a lack of studies on working women in Sri Lanka, the Demographic and Health Survey 2016 indicated inadequate food consumption patterns among women of reproductive age [9]. Studies from India have reported poor dietary patterns or unhealthy eating habits among working women [10,11,12]. Poor diet patterns with a lack of a balanced diet are known to be the primary causes of malnutrition [13]. Earlier studies in different population groups have also shown that adequate nutrition knowledge is an important factor in promoting healthier eating habits [10,14,15]. A study conducted on reproductive-age women living in marginalized areas in Sri Lanka reported a low level of nutritional knowledge and practice about healthy diet among the study participants [16]. However, nutrition knowledge alone may not be sufficient for healthier dietary habits, and hence there is an additional need for a positive attitude toward healthy eating [17,18]. Data on the association between dietary behaviour and knowledge and attitudes among Asian working women are limited [10,19]. Krishna and Dhas in their study showed that working women with higher knowledge and attitudes had better dietary practices [10]. On the contrary, another study from India reported poor dietary behaviour among working women while a large majority of them had a good level of knowledge about a balanced diet [19].

To date, no studies have focused on dietary patterns and/or eating behaviours of working women in Sri Lanka. Further, there is a lack of studies on the nutrition knowledge and attitude towards healthy eating practices of working women in Sri Lanka. As mentioned earlier, a significant proportion of working women in Sri Lanka suffer from malnutrition and chronic diseases, so accessing their dietary patterns and current nutrition knowledge is necessary to develop an appropriate intervention for improving their overall health and nutrition status. Hence, the present study was undertaken to assess the dietary pattern, nutrition-related knowledge, and attitudes towards healthy eating practices of working women in Western Province, Sri Lanka.

## 2. Subjects and Methodology

### 2.1. Study Design and Participants

A cross-sectional study design was utilized to collect data from 300 women aged 20–60 years, who were employed in both public and private organizations in Western Province, Sri Lanka. The working women who were pregnant and lactating were excluded from the study. The study was approved by the Ethical Review Committees of Wayamba University, Sri Lanka and Griffith University, Australia. The study was conducted from January to July 2022.

### 2.2. Sampling

Study participants were selected in two stages: First, both public and private sector organizations were selected from all three districts (Colombo, Gampaha, and Kalutara) of Western Province using a simple random sampling technique. In the second stage, convenience sampling was applied to select an equal number (150) of participants from two sectors, with a total of 300 participants.

### 2.3. Data Collection

The purpose and exact nature of the study were explained to all eligible working women, and those who agreed to participate were requested to sign a consent form before including them in the study. After receiving informed consent, trained interviewers collected the data using a pre-tested structured questionnaire. The questionnaire comprised socio-demographic information, dietary pattern, nutrition-related knowledge, and attitudes.

#### 2.3.1. Socio-Demographic Characteristics

Information on age, marital status, level of education, number of family members, living status, spouse’s education level, and household monthly income were collected.

#### 2.3.2. Dietary Pattern

The information on usual food consumption patterns was assessed by interviewing the participants using a 7-day food frequency questionnaire (FFQ) containing 78 food items that are commonly consumed by the Sri Lankan population. The FFQ questionnaire was adopted from the national nutrition and micronutrient survey [20], modified, and pre-tested in the study population. The food items included in the questionnaire: various grains (rice, bread, and cereals and their products); roots and tubers (sweet potato, manioc and other yams, potato, jak and breadfruit); meat (chicken, beef, pork, mutton, liver and other organ meat, processed meat); fish, shellfish, dry fish, and eggs; pulses (soya meat, cowpea, chickpeas, green grams, black grams, lentils); milk and milk products (milk powder and liquid milk, curd and yoghurt, ice cream, butter, cheese, and ghee); vegetables (pumpkin, carrots, beans, beetroot, cabbage, brinjals, okra, etc.) and seasonal green leafy vegetables (GLV); fruits (banana, papaya, guava, avocado, pineapple, oranges, mango, etc.); nuts and oils, fast food, tea and coffee, sugar-sweetened beverages, fruit juice, and snacks (biscuit, cake, chocolate, etc.). The FFQ was focused on the frequency of intake of selected food items only and information on the portion size was not included. Food frequency intake responses were collected days/week and time/day, then expressed as the total frequency of consumption/week (days/week X time/day).

#### 2.3.3. Dietary Diversity Score

Dietary diversity score (DDS) was assessed using ten food groups based on food group definitions and scoring guidelines recommended by the Food and Agriculture Organization (FAO) for women of reproductive age [21]. All food items included in the FFQ were categorised into 10 food groups as follows: (1) grains, roots and tubers; (2) pulses; (3) nuts and seeds; (4) dairy; (5) meat, poultry and fish; (6) eggs; (7) dark green leafy vegetables; (8) vitamin A rich fruit and vegetables; (9) other vegetables; and (10) other fruits. The DDS was calculated from the data on frequency intake responses collected days/week. The consumption of all food items within a food group was summed up, then divided by seven and expressed as a frequency of consumption of each food group/day. Then, consumption of a food group was given a score of “1” and non-consumption was given a score of “0”, with a maximum score of 10. The DDS was defined as the total number of food groups consumed in a day.

#### 2.3.4. Nutrition-Related Knowledge

The questionnaire was developed by adopting the questions from previous studies [16,22,23], modified, and pre-tested. The nutrition-related knowledge questionnaire contained 16 questions, which addressed topics on a balanced diet, the types and sources of nutrients and some health benefits of the diet. The correct answer for each question received a score of one point and the incorrect one received a score of zero point, so with a maximum score of 16. All answers reported as “don’t know” were coded as incorrect.

#### 2.3.5. Nutrition-Related Attitude

Six questions were included in the nutrition-related attitude questionnaire, which consisted of attitudes on weight status, healthy eating, and behaviours [16,22]. To assess the “attitude” component, a 5-point Likert scale (definitely, probably, probably not, definitely not and don’t know) was used. The 5-point scale was then scored ranging from 1 (don’t know) to 5 (definitely) for answers to all questions, except one that was related to physical activity, where opposite scoring was used (definitely: 1 and don’t know: 5). The maximum possible score was 30.

#### 2.3.6. Anthropometric Assessment

Height was measured with the head in Frankfort horizontal plane and barefoot, to the nearest 0.1 cm using a stadiometer. Weight was measured without shoes to the nearest 0.1 kg using an electronic scale. Body mass index (BMI) was calculated as body weight in kg divided by height in meters squared (kg/m^2^). Then, the participants were classified as underweight (<18.5 kg/m^2^), normal (18.5–25.0 kg/m^2^), overweight (25.0–29.9 kg/m^2^), and obese (≥30.0 kg/m^2^) using the cut-off values recommended by WHO [24].

### 2.4. Statistical Analysis

The statistical analysis was performed using SPSS statistical software version 26 (SPSS Inc., Chicago, IL, USA). The data were presented as frequencies and proportions for all categorical variables and mean, standard deviations, and median for continuous variables. Socio-demographic variables were divided into groups based on prior logical categories. The median value of knowledge scores was used as a cut-off to estimate the proportion of women with good knowledge (knowledge score ≥ median value) and poor knowledge (knowledge score < median value). Similarly, the median value of attitude scores was used as a cut-off to estimate the proportion of women with good attitudes (attitude score ≥ median value) and poor attitudes (attitude score < median value). The relationship between the frequency of consumption of various foods and levels (good vs. poor) of nutrition-related knowledge and attitudes was examined by using an independent *t*-test. The proportion of women meeting the minimum DDS (consumption of 5 out of 10 food groups in a day) was calculated using scoring guidelines recommended by the FAO for women of reproductive age [21]. Pearson’s correlation test was performed to examine the association of DDS with nutritional knowledge and attitudes and various socio-demographic variables. Backward stepwise multiple regression analysis was carried out to examine the independent association of DDS with nutritional knowledge, attitudes, and selected socio-demographic variables. A *p*-value of <0.05 was considered significant in all analyses.

## 3. Results

Table 1 delineates the socio-demographic characteristics of the study participants. The majority (45.7%) of the participants were aged between 31 to 40 years. Of the participants, an equal proportion had either an advanced level or diploma degree, 43% had a bachelor’s degree or above, and only 16% had an ordinary level or below. Nearly 55% of the participants belong to families with four members, and nearly two-thirds (65%) of the women came from nuclear families and lived separately from their parents. Additionally, for their spouses’ education levels, 22.4%, 20.7%, 16.3%, and 40.7% had ordinary level and below, advanced level, diploma, and bachelor’s degree and above, respectively. An almost equal proportion of the participants had either a monthly income between LKR 50,000 to 100,000 (42.6%) or more than LKR 100,000 (40.5%). Based on BMI, 38% of the working women were overweight, 13% were obese, and only 2.7% were underweight. Further, the highest rate of overweight (46.1%) and obesity (19.1%) was observed among working women aged 41–50 years.

The median frequency of intake of chicken, other meat (beef, mutton, pork), organ meat, fish, shellfish, dry fish, eggs, and milk and milk products were two, zero, zero, five, one, three, two, and nine, respectively, per week (Table 2). Thirty-five per cent of the women had chicken, 30.6% had eggs, 69.7% had fish, and 57.7% had milk and milk products at least four times in the week preceding the interview (Table 2). The median frequency of intake of GLV, other vegetables and fruits were five, ten, and ten, respectively, per week (Table 2). Two-thirds (67%) of the women consumed GLV and 82% had fruits at least four times a week. On the other hand, 80% of the women never had other meat (beef, mutton, or pork), 90% never had liver or any other organ meat, and 23% never had milk and milk products. A large majority of the women never had shellfish (prawns, crabs, shrimps; 47%), processed meat (43%), fast food (46%), fruit juice (45%) and sugar-sweetened beverages (71%), while over 60% of women had tea and coffee with sugar seven times or more per week.

**Figure 1:**
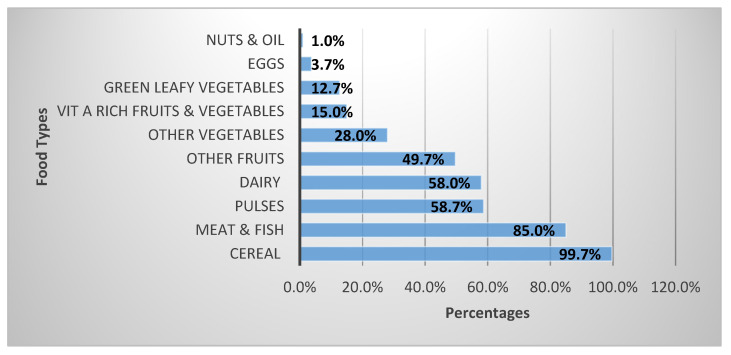
Consumption of different food groups by working women on average once a day over one week.

Figure 1 shows the consumption of different food groups by the participants at least one time per day over one week. Almost all women had consumed grains, roots, and tubers. Eighty-five per cent of the women had meat, poultry, and fish. On the other hand, a large majority of the women did not consume nuts and seeds (99%), eggs (96%), vitamin A-rich fruit and vegetables (85%), GLV (87%), other vegetables (72%), and other fruits (50%). Over 40% of the women did not consume dairy and pulses. The mean (±SD) DDS was 4.12 (±1.49), with a median (range) of 4.0 (1–9). Nearly two-thirds (65%) of the working women did not meet the minimum DDS.

Table 3 depicts the proportion of working women who correctly answered nutrition-related knowledge questions. Over 80% of the working women answered correctly about a balanced diet. More than 95% of the women knew about good sources of protein (98.7%), carbohydrates (96%), and fat (98%). Over 80% of the women knew about the foods that are rich in iron, the foods that can increase and/or decrease iron absorption, and about health consequences of low intake of iron-rich foods. Over half (53.3%) of the women articulated that the importance of fruit and vegetables is only due to their vitamin and mineral contents. Around 70% of the women incorrectly stated that vitamins as a good source of energy and 60% reported skipping a meal is a good way to lose weight. Nearly half (45%) of the women did not know that oily fish contains healthier fat than red meat fat. A similar proportion (44.3%) also did not know that obesity is associated with chronic diseases such as heart disease. The mean (±SD) nutrition-related knowledge score was 11.95 (±2.04), with a median (range) of 12.0 (2–16). About sixty (59.7%) per cent of the women had good knowledge (knowledge score ≥ 12), while the rest (40.3%) had poor knowledge (knowledge score < 12).

The study participants were asked about their feelings towards their current weight status, healthy eating, and behaviours (Table 4). Over a quarter (28.3%) of the women stated that their current body weight is harmful to their health and nearly 40% of women said they were motivated to lose weight. Twenty-one per cent of the women said they are not satisfied with their current level of physical activities, while another 28% said they are satisfied. A quarter of the study participants said small and frequent meals help in weight reduction. A large majority (88%) of the working women said eating breakfast is part of a healthy lifestyle, while 36% of the women said eating a mixed diet regularly is a healthy eating behaviour. The mean (±SD) nutrition-related attitude score was 21.8 (±3.50), with a median (range) of 22.0 (13–29). Overall, 56.0% of the participants had good attitudes (attitude score ≥ 22) and the rest of the 44.0% had poor attitudes (attitude score < 22).

Table 5 shows the relationship of food consumption patterns with nutrition-related knowledge and attitudes of working women. The women with good nutrition-related knowledge had a significantly higher frequency of consumption of roots/tubers (*p* = 0.005), shellfish (*p* = 0.014), vegetables (*p* = 0.044), fruits (*p* = 0.020), fruit juice (*p* = 0.029), nuts and oils (*p* = 0.022), and fast food (*p* = 0.034), while they had a significantly lower frequency of consumption of chicken (*p* = 0.028). Women with good nutrition-related attitudes had a significantly lower frequency of consumption of soya meat (*p* = 0.017) but a higher frequency of consumption of fast food (*p* = 0.022). None of the other food consumptions were related to nutrition-related attitudes.

The association of DDS with nutrition-related knowledge, attitudes, and various sociodemographic variables were tested using Pearson’s correlation. There were significant positive associations between the DDS and age of the participants (r = 0.157; *p* = 0.006), education level of the participants (r = 0.214; *p* = 0.000), spouse’s education level (r = 0.202; *p* = 0.000), household income (r = 0.273; *p* = 0.000), and nutrition-related knowledge (r = 0.159; *p* = 0.006). On the other hand, the dietary diversity score was negatively associated with the duration of work/week (r = −0.209; *p* = 0.000). Factors influencing the DDS of the working women were evaluated using a backward stepwise multiple regression analysis (Table 6). When age, level of education, duration of work (hours/week), spouse’s education level, household income, and nutrition-related knowledge and attitudes were included in the analysis and, using a *p* = 0.10 for the exclusion level, education, duration of work (hours/week), spouse’s education level dropped out of the model. Among the variables left in the equation, age, and household income were significantly independently related to DDS, while nutrition-related attitudes were negatively associated. There was a positive independent association between DDS and nutrition-related knowledge; however, it did not reach statistical significance (*p* = 0.057). The household income bore a stronger association with DDS compared to other variables judged by the comparable beta coefficient. The overall F ratio was 8.46 (df = 4) and was highly significant (*p* = 0.001). The adjusted R square was 0.093 (multiple R = 0.325), suggesting the variables in the equation accounted for a 9.3% variance in DDS.

## 4. Discussion

This study provides insight into the nutritional status, dietary pattern and nutrition-related knowledge and attitudes towards healthy eating of working women in Western Province, Sri Lanka. To our knowledge, this is the first study that focused on working women in Sri Lanka. Overall, most of the participants came from educated families and relatively higher social positions and better economic conditions.

Over half of the working women were either overweight (38%) or obese (13%), whereas only a few (2.7%) were underweight. While there are no data on working women, the Sri Lankan Demographic and Health Survey (DHS) 2016 reported a 32% of prevalence of overweight and a 13% prevalence of obesity among ever-married women aged 15–49 years [9], which is very similar to our study findings.

The data on usual dietary patterns, based on 7-day FFQ, revealed a wide variation in intake patterns. First, comparing the frequency of intake of the foods of animal origin each week, fresh fish was most frequently consumed, followed by dry fish, chicken, and eggs. Looking at the distribution of the frequency of consumption, nearly 70% of the working women had fresh fish and 43% had dry fish at least four times a week. Both fresh fish and dry fish are good sources of protein in the Sri Lankan diet. Additionally, fish, particularly oily fish, contain omega-3 fatty acids and regular consumption of fish may help reduce the risk of heart disease and stroke [25]. Over two-thirds of the working women had eggs only occasionally (one-to-three times a week) or no eggs at all. Eggs are a very good source of protein and some vitamins, such as vitamins A, D, and B_12_. Quite surprisingly, red meat such as mutton, pork, beef, and organ meat were not so popular—over 80% of the working women did not consume these foods at all in the week preceding the interview. While there is a lack of data on the detailed food consumption patterns of Sri Lankan women, a study conducted among rural adults in dry-zone Sri Lanka also reported a very similar eating pattern with 54% having fish, 12.5% having eggs, and only 7.5% having meat and meat products, and there was no significant difference in the mean consumption pattern between males and females [26]. However, this study assessed the food consumption pattern using 24 h dietary recall method. Of note, a low intake of red meat and organ meat is a healthy eating habit, as regular consumption of larger quantities of red meat may be associated with an increased risk of colorectal cancer [27] and is associated with increased consumption of saturated fat—unless it is lean meat—which is a known risk factor for cardiovascular disease [28].

The food consumption data also reveal that the intake of pulses is reasonably high, which 85% of the women consumed at least four times a week. While not directly comparable, the Sri Lankan DHS 2016 reported, based on 24 h dietary recall, that over two-thirds of the women of reproductive age had legumes/pulses on the day preceding the interview [9]. Pulses are a good source of plant protein and fibre, as well as a significant source of vitamins and minerals, such as iron, zinc, folate, and magnesium, and regular consumption of pulses is associated with reduced risk of several chronic diseases [29].

In the present study, we found that one in four working women did not consume milk and milk products at all and another 20% of the women had it occasionally. The Sri Lankan DHS 2016 also showed that only 20% of the women had milk and yoghurt on the day preceding the interview [9]. Dairy products contain vitamin D and other minerals for instant calcium and phosphorus which are important for the maintenance of bone health and reducing fractures [30,31].

The intake of other vegetables and fruits was reasonably high among the study participants with a median consumption of 10 times/week. Further, our data showed more than 80% of women had other vegetables and fruits at least four times per week. However, one-third of the women had GLV only occasionally, i.e., one-to-three times a week or not at all. These foods are nutrient-dense, relatively low in energy, and are good sources of minerals and vitamins, dietary fibre, and a range of phytochemicals including carotenoids [32]. The health benefits of consuming diets high in vegetables and fruit have been reported for decades [33,34].

Almost all the participants (99%) regularly consumed grains/cereals with a mean consumption of 20 times/week, which is expected as rice is the staple food in Sri Lanka. Further, roots and tubers (mostly starchy vegetables) intake was also relatively high with a mean consumption of 4.5 times a week. Typical Sri Lankan cuisine contains mainly a larger portion of boiled or steamed rice with a curry of fish, meat, or egg along with other curries made with lentils (pulses), starchy vegetables such as potato, breadfruit, jackfruit, or manioc—a predominantly carbohydrate diet. The Sri Lankan DHS 2016 also reported that 96% of the women had grains and 55% had roots and tubers on the day preceding the interview [9].

In the present study, we observed a mean DDS of 4.12 based on the consumption of different food groups at least one time, irrespective of portion size, per day over one week. Looking at the findings of DDS, a large majority of the women did not consume eggs (96%), vitamin A-rich fruits and vegetables (85%), or GLV (87%) at least one time a day in the week preceding the survey. Additionally, nearly half of the women did not consume other fruits, dairy, and pulses. Similar to the findings of our study, Abeywickrama et al. in their study reported that only 10% of the rural adults in dry-zone Sri Lanka had any fruit, and around 40% had GLV [26]. The Sri Lankan DHS 2016 also reported that 88% of the women of reproductive age did not consume vitamin A-rich fruits and vegetables, 50% had no other fruits, and 69% had no legumes/pulses on the day preceding the interview [9]. However, Abeywickrama et al. and the DHS 2016 reported the consumption of various food groups based on 24 h dietary recall method [9,26]. Moreover, the food groups used were also not comparable with our study, except a few. We also estimated the proportion of women meeting the minimum DDS according to FAO guidelines [21], which indicates micronutrient adequacy in the diet [35]. Nearly two-thirds of the women did not meet the minimum DDS, indicating that they are unlikely to meet their daily micronutrient needs.

Processed meat, fast food, and snacks are classified as discretionary choices because they are energy-dense and high in saturated fat, trans fat, and/or salt. Regular consumption of these foods is known to be associated with an increased risk of becoming overweight or obese, which can lead to the development of several chronic diseases [36]. In the present study, we found that about half of the women had processed meat (49%) or fast food (50%) one-to-three times a week preceding the interview, while the consumption of snacks was very high with a mean intake of 4.8 times/week. A study conducted among working mothers of preschool children in Mumbai, India also reported high consumption of fast food [11]. Like the findings of the present study, a study conducted among working women in Jabalpur, India has also reported the habit of taking snacks in a regular pattern [12]. Another study from Odisha, India also reported that two-thirds of the working women had snacks once a day [37]. Our data also showed that nearly two-thirds of the working women consumed tea and coffee with sugar seven times or more per week. Nearly half of the working women had fruit juice and 27% of the women had sugar-sweetened beverages one-to-three times a week. Consumption of added sugars has been associated with an increased risk of obesity [38] as well as increased risk factors for developing diabetes [39].

According to the knowledge, attitude, and practice theory, the process of human behaviour change occurs in three steps: acquiring knowledge, generating attitudes/beliefs, and forming practice/behaviours [40]. The present study revealed that more than half of the working women had good basic nutrition-related knowledge and favourable attitudes to weight status and healthy eating. A study conducted among reproductive-age women in marginalized areas in Sri Lanka showed that the average knowledge score of employed women was higher than unemployed women [16].

We sought to explore the relationship between the frequency of food consumption patterns and nutrition-related knowledge and attitudes of working women. Compared to women with poor nutrition-related knowledge, a significantly higher frequency of intake of roots/tubers, shellfish, nuts and oils, vegetables, fruit, and fruit juice was observed in the women with good nutrition-related knowledge. Our study findings are consistent with the findings of the previous studies. Wardle et al. in their study among adults in England have reported a significant association between knowledge and consumption of fruit and vegetables [41]. In a systematic review, Spronk et al. also found that most of the studies that were included in the analysis reported an association between higher knowledge and higher intake of fruit and vegetables [42]. While we observed a positive association between knowledge and attitudes, nutrition-related attitudes did not translate into healthy eating patterns, i.e., we did not find any association of attitudes with fruit and vegetable consumption. In this study, we found that women with good nutrition-related attitudes had a significantly lower frequency of consumption of soya meat only. Surprisingly, we found a significantly higher frequency of consumption of fast food among women with good knowledge and good attitudes, whereas one would expect the opposite relationship. Of note, covariate analyses showed that family income was a mediator of fast food consumption, which may partially explain the present finding.

In the present study, we also identified factors associated with DDS in working women. Our findings revealed that age and household monthly income were significantly independently associated with DDS. Household income, an indicator of socio-economic status, has been consistently found to be associated with the consumption of a better-quality diet. For example, a study among US adults suggested that a better socioeconomic status index is independently associated with adequate fruit and vegetable intake and overall diet quality [43]. Another study from Iran also showed that families with good socio-economic status consumed significantly more fruit, vegetable, dairy, red meat, chicken and poultry, fish, and egg [15]. The positive association between age and DDS could be due to the relatively better nutrition knowledge among older women, and their ability to make better food choices. Notably, we observed a positive correlation between age and nutrition-related knowledge. Similarly, previous studies have shown that older women have better nutrition knowledge [44]. Earlier studies have shown that nutrition-related knowledge is associated with a healthy eating pattern [15,44,45]. In the present study, we found a trend of a marginally significant positive association between nutrition-related knowledge and DDS (*p* = 0.057). In this study, nutrition-related attitudes were negatively associated with DDS. The reason for this unexpected finding could be due to the content of the attitudes questions not well capturing the dietary diversity but rather focusing on weight status. Further studies are warranted to confirm the association between attitudes and DDS.

The present study has some limitations. First, this study was conducted on a convenience sample; thus, the study findings may not be representative of the wider population, so the results should be interpreted with caution. Second, the sample size was relatively small considering the nature of the data, which may have introduced some bias. Third, the lack of data on portion size limits information regarding the actual consumption of energy and nutrient intake. Forth, data obtained using a cross-sectional survey did not allow us to determine the causality.

One of the strengths of our study is that the DDS calculation was based on the average daily consumption of any food groups over a reference period of the previous 7 days. Thus, this method is likely to give more accurate and reliable estimates of the diversity of diet than a DDS calculated using a single 24 h recall. The present study used multivariate analysis to identify the potential determinants of DDS by controlling the effect of confounders in this population group. In addition, this study, for the first time, provided a snapshot of the dietary pattern, nutrition-related knowledge, and attitudes of working women in Sri Lanka and their association, which can help design further research to improve the overall situation of this population group.

## 5. Conclusions

The results demonstrate that a significant proportion of working women in the Western Province of Sri Lanka have good levels of basic nutrition-related knowledge and attitudes about their current weight status, healthy eating, and behaviours. Women with good nutrition-related knowledge are more frequent consumers of roots/tubers, shellfish, vegetables, fruit, fruit juice, nuts and oils, and fast food. A large majority of the women do not meet the minimum DDS, indicating that they face substantial risk regarding micronutrient deficiencies. Furthermore, age, family income and nutrition-related knowledge were positively associated with DDS among working women, but a negative association was found between DDS and nutrition-related attitudes. Before designing any intervention, further research is needed using a qualitative approach to understand how nutrition knowledge and eating behaviour are related in this population group.

## Figures and Tables

**Table 1:** Socio-demographic characteristics of the study participants.

Variable	*n*	%
Age Group (Year)		
20 to 30	40	13.3
31 to 40	137	45.7
41 to 50	89	29.7
51 to 60	34	11.3
Marital Status		
Married	291	97.0
Unmarried/Divorced/Widowed	9	3.0
Participant’s Education Level		
Ordinary level and below	49	16.3
Advanced level	61	20.3
Diploma	61	20.3
Bachelor’s degree and above	129	43.0
Family Size		
Up to three members	136	45.3
Four members and above	164	54.7
Living Status		
Extended family	105	35.0
Nuclear family	195	65.0
Spouse’s Education Level *		
Ordinary level and below	66	22.4
Advanced level	61	20.7
Diploma	48	16.3
Bachelor’s degree and above	120	40.7
Household Income ** (LKR ***/month)		
<50,000	50	16.9
50,001 to 100,000	126	42.6
100,000 and above	120	40.5

* Data missing for 5 participants; ** Data missing for 4 participants. *** USD 1 = LKR 370.

**Table 2:** Mean (SD) and median frequency of intake, and frequency distribution of various food items consumed, during the past week by the study participants.

				Frequency of Consumption, Times/Week			
Food Item	Mean (SD)	Median	Range	Never %	1–3 %	4–6 %	≥7 %
Grains	20.2 (5.7)	20.0	3–39	0	0.7	0.3	99.0
Roots and tubers	4.5 (3.7)	4.0	0–25	8.3	40.3	32.3	19.0
Chicken	3.2 (2.8)	2.0	0–18	13.3	51.7	23.3	11.7
Other meat	0.4 (1.1)	0.0	0–8	80.0	17.3	1.7	1.0
Organ meat	0.1 (0.4)	0.0	0–21	89.7	10.3	0	0
Processed meat	1.1 (1.3)	1.0	0–8	43.3	49.3	7.0	0.3
Fish	5.7 (4.1)	5.0	0–21	7.7	22.7	36	33.7
Dried fish/sprats	3.5 (3.1)	3.0	0–21	10.7	46	32.7	10.7
Shellfish	1.0 (1.9)	1.0	0–21	47.0	47.7	4.0	1.2
Egg	3.1 (3.0)	2.0	0–21	6.0	63.3	12.3	18.3
Soya meat	1.4 (2.0)	1.0	0–15	40.0	50.0	7.0	3.0
Pulses	9.2 (5.6)	8.0	0–33	0.7	11.7	25.3	62.3
Milk and milk products	10.4 (6.9)	9.0	0–38	23.0	19.3	6.7	51.0
Vegetables	11.7 (6.3)	10.0	0–31	0.3	5.7	14.3	79.7
Green leafy veg	5.6 (3.8)	5.0	0–21	4.0	29.0	30.7	36.3
Fruits	10.3 (7.2)	10.0	0–43	3.0	15.0	13.3	68.7
Nuts	1.0 (1.4)	1.0	0–14	44.3	51	3.3	1.3
Fast food	0.9 (1.4)	1.0	0–14	46.0	50.0	2.3	1.7
Snacks	4.8 (3.5)	4.0	0–21	4.7	34.7	30.3	30.3
Tea/coffee with sugar	7.8 (6.0)	7.0	0–21	14.7	14.7	7.7	63.0
Sugar-sweetened beverages	0.5 (1.1)	0.0	0–14	71.0	27.0	1.7	3.0
Fruit juice	1.0 (1.6)	1.0	0–14	45.0	49.3	4.0	1.7

**Table 3:** Nutrition-related knowledge of the study participants.

Question	Correct Answer	
	*n*	%
A balanced diet implies eating all food groups in the same amount	248	82.7
Which food is a rich source of carbohydrate	288	96.0
Which food is rich in protein	296	98.7
Which food is rich in fat	294	98.0
Which food is high in fibre	267	89.0
Which food is rich in vitamins	297	99.0
Which food rich in iron	256	85.3
Foods that increase iron absorption	245	81.7
Drink that decreases iron absorption	264	88.0
Vitamins are a good source of energy	91	30.3
Oily fish (Mackerel, Salmon) contains healthier fats than red meat	165	55.0
The health benefit of fruits and vegetables are only due to their vitamins and minerals contents.	140	46.7
For healthy nutrition, dairy products should be consumed in the same amount as fruits and vegetables	184	61.3
A lack of iron in the diet can result in fatigue and illness	261	87.0
Obesity is associated with heart diseases	167	55.7
Skipping meals is a good way to lose weight	121	40.3

**Table 4:** Nutrition-related attitudes of the study participants.

Statement	Definitely %	Probably %	Probably Not %	Not %	Don’t Know %
I consider my current weight to be harmful to my health	28.3	27.7	12.7	27	4.3
I am motivated to lose weight	39.0	28.3	9.3	23.3	0
I consider regular breakfast intake to be a part of a healthy lifestyle	87.7	10.3	0.7	1.3	0
I consider small and frequent meals to help in weight reduction	24.0	27.0	17.7	27.3	4.0
Eating a variety of food items each day is a healthy option	36.0	37.0	16.0	7.0	4.0
I am satisfied with my current physical activity level	28.3	33.0	17.3	21.3	0

**Table 5:** Relationship between the frequency of consumption of various foods and nutrition-related knowledge and attitudes of the study participants.

	Knowledge			Attitudes		
	Poor, *n* = 121	Good, *n* = 179		Poor, *n* = 132	Good, *n* = 168	
Food Item	Mean (SD)	Mean (SD)	*p*-Value	Mean (SD)	Mean (SD)	*p*-Value
Grains	18.5 (5.6)	18.6 (5.0)	0.847	18.3 (5.2)	18.8 (5.3)	0.436
Roots and tubers	5.2 (3.8)	6.6 (4.8)	0.005	5.5 (4.1)	6.5 (4.8)	0.066
Chicken	3.6 (3.4)	2.8 (2.4)	0.028	3.0 (2.7)	3.2 (3.0)	0.541
Other meat	0.40 (1.1)	0.35 (1.1)	0.726	0.39 (1.0)	0.35 (1.1)	0.729
Organ meat	0.12 (0.4)	0.12 (0.3)	0.883	0.15 (0.4)	0.10 (0.3)	0.208
Processed meat	1.16 (1.4)	0.99 (1.3)	0.298	1.10 (1.3)	1.02 (1.3)	0.633
Fish	5.6 (3.8)	5.8 (4.3)	0.655	5.5 (3.7)	5.9 (4.4)	0.308
Dried fish/sprats	3.6 (3.3)	3.4 (3.0)	0.667	3.5 (3.0)	3.5 (3.3)	0.967
Shellfish	0.73 (1.3)	1.22 (2.2)	0.014	0.83 (1.2)	1.17 (2.3)	0.087
Egg	3.1 (2.6)	3.1 (3.3)	0.984	3.1 (2.9)	3.1 (3.1)	0.964
Soya meat	1.4 (2.3)	1.3 (1.9)	0.616	1.7 (2.5)	1.1 (1.6)	0.017
Pulses	9.6 (5.7)	8.9 (5.6)	0.321	9.1 (5.4)	9.3 (5.8)	0.745
Milk and milk products	10.1 (6.9)	10.6 (6.9)	0.507	10.5 (7.3)	10.3 (6.7)	0.759
Vegetables	10.8 (6.0)	12.2 (6.4)	0.044	11.5 (6.2)	11.8 (6.4)	0.693
Green leafy veg	5.5 (3.8)	5.7 (3.7)	0.699	5.6 (3.5)	5.6 (4.0)	0.968
Fruits	9.2 (6.7)	11.1 (7.4)	0.020	9.9 (7.8)	10.7 (6.6)	0.341
Nuts and oils	0.74 (1.1)	1.1 (1.6)	0.022	0.84 (1.5)	1.04 (1.4)	0.246
Fast food	0.72 (1.2)	1.1 (1.6)	0.034	0.72 (1.0)	1.08 (1.7)	0.022
Snacks	4.6 (3.3)	5.0 (3.6)	0.410	4.5 (3.3)	5.1 (3.7)	0.106
Tea/coffee with sugar	7.1 (5.6)	8.2 (6.2)	0.108	8.2 (6.0)	7.5 (6.0)	0.283
Sugar-sweetened beverages	0.40 (0.7)	0.51 (1.3)	0.333	0.39 (0.8)	0.52 (1.3)	0.300
Fruit juice	0.80 (1.1)	1.2 (1.8)	0.029	0.89 (1.2)	1.1 (1.8)	0.159

**Table 6:** Multiple linear regressions for dietary diversity score of the study participants.

Model	B	s.e. B	Beta	*p*-Value
Age	0.024	0.010	0.138	0.020
Household income	0.504	0.121	0.245	0.001
Knowledge Score	0.080	0.042	0.110	0.057
Attitude Score	−0.051	0.025	−0.120	0.045

R^2^ = 0.105; Adjusted R^2^ = 0.093; F = 8.46 (df = 4); *p* value = 0.001.

## Data Availability

The data presented in this study are available on request from the corresponding author. The data are not publicly available due to ethical restrictions.
